# Resolution of the Mad River Valley Association

**Published:** 1866-02

**Authors:** 


### RESOLUTION OF THE MAD RIVER VALLEY DENTAL SOCIETY.

*Resolved*, That this Association is appreciative of the valuable services of Dr. J. Taft, Editor of the Dental Register of the West, rendered the profession, in continuing, ably and progressively, its publication during the recent crisis of our country, and now continuing, so interestingly and hopefully, its publication; and that it deserves the liberal patronage of every honorable practitioner, in our rising Profession.

Ed. Register.—The above resolution, passed at the summer meeting of the Association, was handed to me to transmit to you; but I unfortunately lost it. To-day I found it; and believing it to be, as it was then, the unanimous sentiment of the Association, it is now at your service. Our Society's next meeting will be at Dayton, the 6th of March. Come up and see us.

